# Human leukocyte antigen (HLA) class II peptide flanking residues tune the immunogenicity of a human tumor-derived epitope

**DOI:** 10.1074/jbc.RA119.009437

**Published:** 2019-10-16

**Authors:** Bruce J. MacLachlan, Garry Dolton, Athanasios Papakyriakou, Alexander Greenshields-Watson, Georgina H. Mason, Andrea Schauenburg, Matthieu Besneux, Barbara Szomolay, Tim Elliott, Andrew K. Sewell, Awen Gallimore, Pierre Rizkallah, David K. Cole, Andrew Godkin

**Affiliations:** ‡ Division of Infection and Immunity and Systems Immunity Research Institute, Cardiff University, Cardiff CF14 4XN, United Kingdom; § Institute of Biosciences and Applications, NCSR “Demokritos,” Agia Paraskevi, 15341 Athens, Greece; ¶ Institute for Life Sciences, University of Southampton, Southampton SO17 1BJ, United Kingdom; ‖ Centre for Cancer Immunology, University of Southampton, Faculty of Medicine, University Hospital, Southampton SO16 6YD, United Kingdom; ** Department of Gastroenterology and Hepatology, University Hospital of Wales, CF14 4XN Cardiff, United Kingdom

**Keywords:** T-cell biology, tumor immunology, antigen presentation, molecular dynamics, crystallography, structure-function, antigen recognition, peptide flanking residues

## Abstract

CD4^+^ T-cells recognize peptide antigens, in the context of human leukocyte antigen (HLA) class II molecules (HLA-II), which through peptide-flanking residues (PFRs) can extend beyond the limits of the HLA binding. The role of the PFRs during antigen recognition is not fully understood; however, recent studies have indicated that these regions can influence T-cell receptor (TCR) affinity and pHLA-II stability. Here, using various biochemical approaches including peptide sensitivity ELISA and ELISpot assays, peptide-binding assays and HLA-II tetramer staining, we focused on CD4^+^ T-cell responses against a tumor antigen, 5T4 oncofetal trophoblast glycoprotein (5T4), which have been associated with improved control of colorectal cancer. Despite their weak TCR-binding affinity, we found that anti-5T4 CD4^+^ T-cells are polyfunctional and that their PFRs are essential for TCR recognition of the core bound nonamer. The high-resolution (1.95 Å) crystal structure of HLA-DR1 presenting the immunodominant 20-mer peptide 5T4_111–130_, combined with molecular dynamic simulations, revealed how PFRs explore the HLA-proximal space to contribute to antigen reactivity. These findings advance our understanding of what constitutes an HLA-II epitope and indicate that PFRs can tune weak affinity TCR–pHLA-II interactions.

## Introduction

CD4^+^ T-cells orchestrate immune responses to antigens through the binding of highly variable αβ T-cell receptors (TCRs)^5^ to peptide epitopes presented on major histocompatibility class II (MHC-II or in humans HLA-II) molecules. Such peptide epitopes are often derived from internalized proteins that are digested to short peptides in a specialized endocytic antigen-processing pathway (reviewed in Ref. 1). HLA-II molecules loaded with such peptides (pHLA-II) are mainly expressed by professional antigen-presenting cells (APCs), which sample the periphery for extracellular antigens of potential immunogenicity. Through this pathway, CD4^+^ T-cells have been shown to play an important role in tumor surveillance (2).

Due to the open-ended nature of the peptide-binding groove of HLA-II, compared with the closed groove of HLA-I, loaded peptide epitopes can vary in length typically between 12 and 20 amino acids (3). Thus, outside of the 9-amino acid “core” binding region, HLA-II peptides can extend out of the binding groove, forming peptide-flanking residues (PFRs). Structurally, based on the limited available data, PFRs tend to continue a linear extension out of the HLA-II–binding groove for 1 to 2 residues (reviewed in Ref. 4), whereas longer PFRs can extend away from the HLA surface (5) and form a secondary structure (6).

Murine models have shown the presence of PFRs influences peptide–MHC-II stability (7), T-cell activation (8), TCR gene usage (9), and TCR specificity (10). In humans, processed peptide antigens eluted from HLA-II frequently contain PFRs (3, 11), are recognized by human CD4^+^ T-cells (6, 12) and can be utilized to enhance TCR binding affinity through modification (13). Molecular understanding of how PFRs may enhance HLA-II and/or TCR binding is limited, which makes defining HLA-II–restricted epitopes challenging compared with HLA-I–restricted epitopes (14).

The role of PFRs during CD4^+^ T-cell recognition of cancer epitopes has not been well-defined and may represent an opportunity to design optimized peptides for vaccine or other therapeutic approaches. Here, we focused on understanding how PFRs might influence CD4^+^ T-cells to the oncofetal antigen 5T4, which is up-regulated in a number of epithelial-derived cancers (15) including colorectal cancer (CRC) (16). We have shown 5T4-specific CD4^+^ T-cells are associated with better control of CRC *in situ* (17) and that vaccine-boosted 5T4-specific T-cells lead to improved survival of patients (18).

We have previously examined the functional characteristics of HLA-DRα*0101 and HLA-DRβ1*0101 (henceforth DR1)-restricted CD4^+^ T-cells recognizing influenza A virus (IAV) hemagglutinin (13, 19). To compare characteristics, we isolated three human HLA-DR1–restricted CD4^+^ T-cell clones that recognized regions of 5T4 that generate T-cell responses across multiple subjects (20). Through cellular analyses on these HLA-DR1–restricted T-cell clones, we investigate the recognition profile of different regions of the 5T4 protein. By combining structural analysis with cellular screening of peptide truncations/substitutions, we further dissect the immunogenicity of specific regions within a 20-mer 5T4 peptide epitope. Here, we show PFRs enhance peptide-HLA binding and activation of a cognate T-cell clone and use molecular dynamics simulations to explore how PFRs may enhance immunogenicity. Together, we show PFRs play a fundamental role in driving T-cell activation, supporting the notion that manipulating PFRs may generate more effective therapeutic anti-cancer immunity.

## Results

### CD4^+^ T-cell clones selected against immunodominant 5T4 epitopes exhibit relatively low sensitivity

**Figure 1.**
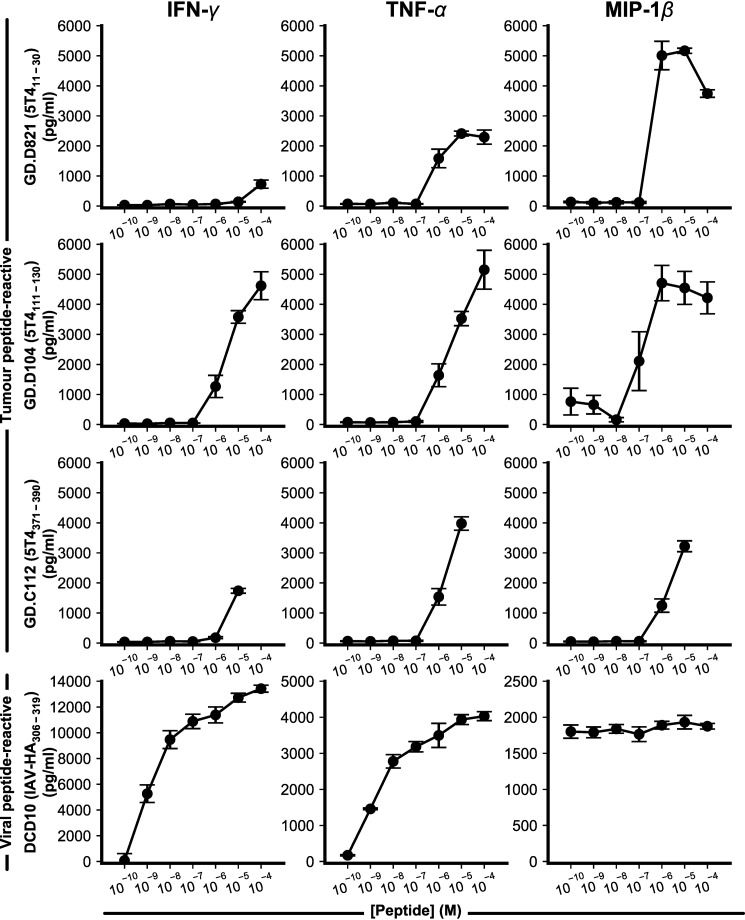
**Impaired peptide sensitivity of 5T4-reactive CD4^+^ T-cell clones.** Soluble IFN-γ, TNFα, and MIP-1β release by three 5T4-responsive CD4^+^ T-cell clones and a representative influenza A virus hemagglutinin (*IAV-HA*)-responsive CD4^+^ T-cell clone. Cytokine/chemokine measured by ELISA in response to overnight co-culture with T2-DR1–presenting cells and titrated cognate peptide. Representative examples (mean ± S.D.) of two independent experiments were performed in at least duplicate is shown.

CD4^+^ T-cell responses to 5T4-derived peptide antigens have been detected in the periphery of healthy donors and patients with CRC (17) where peptide epitopes presented by HLA-II alleles have been mapped (20). To characterize further this response to 5T4, we generated CD4^+^ T-cell clones from an HLA-DR1^+^ donor to three mapped immunodominant 5T4-derived 20-mers. These clones were selected on function (IFN-γ^+^) in response to peptide presented by T2 cells (21) transduced with HLA-DR1 (T2-DR1) (22). CD4^+^ T-cell clones reactive to three epitopes were obtained: 5T4_11–30_-reactive (GD.D821), 5T4_111–130_-reactive (GD.D104), and 5T4_371–390_-reactive (GD.C112) (Fig. 1).

Each clone responded in T-cell activation assays by producing IFN-γ and TNFα in response to peptide where one or both cytokines were detectable down to 10^−6^ m concentration of peptide. Each clone also produced the early marker of activation MIP-1β (CCL4) down to 10^−6^-10^−7^ m peptide. Although each 5T4 cancer antigen-reactive clone exhibited similar reactivity, this sensitivity was markedly inferior (∼100-fold weaker) to CD4^+^ T-cell clones recognizing pathogen-derived epitopes (representative example to IAV hemagglutinin_306–319_ epitope shown). Despite this far weaker sensitivity, cross-recognition of irrelevant 5T4 peptides was not detected (Fig. S1).

### CD4^+^ T-cell clone reactivity can occur despite undetectable TCR binding

**Figure 2.**
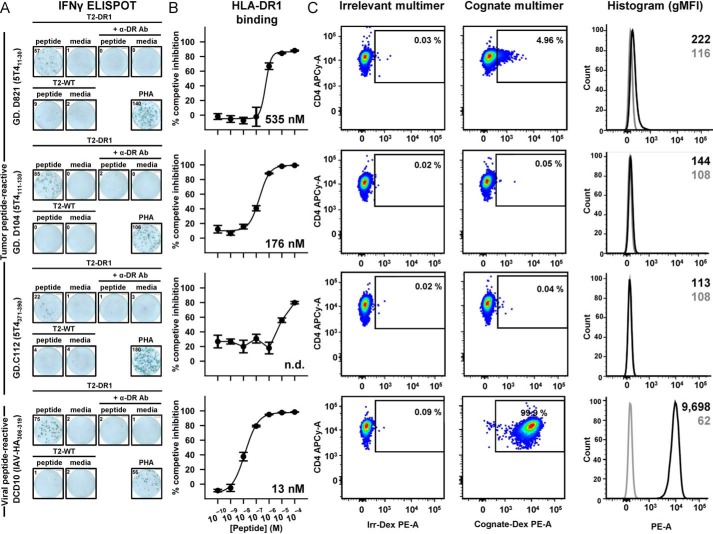
**5T4 clone reactivity to HLA-DR1–presented peptides despite no measurable ligand engagement.** *A,* IFN-γ ELISpot assays of clones in response to overnight co-incubation with peptide-pulsed APCs. IFN-γ release was observed using DR1-only (*T2-DR1*) but not DR1-null (*T2-WT*) presenters in the presence of peptide but not no-peptide (media) controls. IFN-γ release was blocked by an αDR blocking antibody (+α-*DR Ab*). GD.D821, GD.D104, GD.C112, and DCD10 were stimulated with APCs pulsed with 10^−5^, 10^−6^, 10^−5^, and 10^−6^ m peptide, respectively. Maximal IFN-γ response indicated by phytohemagglutinin (*PHA*) activation. *Inset numbers* represent raw spot forming cells (*sfcs*). Presented ELISpot wells representative of two duplicate experiments. *B,* binding capacity of each peptide to HLA-DR1 molecules in competitive binding assays *in vitro. Error bars* indicate S.D. of experiments performed in triplicate. *Inset number* denotes IC_50_ value calculated from displayed curve fit. N.D. = IC_50_ not determined. *C,* cognate HLA-DR1 multimer staining of each 5T4-reactive clone exhibiting staining marginally above background (irrelevant multimer). This was in stark contrast to typical staining of the DCD10 viral-reactive clone. Histogram representation displays inset geometric mean fluorescent intensity of cognate -DR1 multimer (*black*) and irrelevant -DR1 control (*gray*).

To identify the HLA restriction of each clone, low T-cell numbers (300 T-cells) were stimulated overnight with peptide plus various APCs and assayed by IFN-γ ELISpot. Each clone was responsive to tumor-derived peptide presented by a autologous (HLA-II^+^) B lymphoblastoid cell line (data not shown) or T2-DR1 cells in co-culture IFN-γ ELISpot assays (Fig. 2*A*). This reactivity was inhibited by anti–HLA-DR blockade and no such activation was observed using T2-WT (HLA-II^−^) as presenting cells. Assessment of *in vitro* HLA-DR1 binding showed two of the 5T4 peptides (5T4_11–30_ and 5T4_111–130_) bound to HLA-DR1 at reasonable affinities (IC_50_ = 535 and 176 nm, respectively) although weaker compared with the universal IAV epitope HA_306–319_ (IC50 = 13 nm) suggesting that peptide-HLA binding may at least in part affect cognate clone sensitivity (Fig. 2*B*).

We attempted to detect GD.D821, GD.D104, and GD.C112 engagement of cognate HLA-DR1 presented ligand via enhanced multimer staining techniques previously used to isolate weak avidity T-cell clones (23–25). Despite these protocols, detectable engagement of HLA-DR1–5T4 multimers to GD.D821, GD.D104, and GD.C112 cells was repeatedly barely above background, with only GD.D821 exhibiting limited staining compared with irrelevant controls (Fig. 2*C*). This was despite high surface TCR expression by all 5T4-reactive clones (Fig. S2). Such detection was in stark contrast to IAV-specific clones using the same methodology (Fig. 2*C* and Ref. 13). Thus, despite peptide specificity, detectable engagement of cognate ligand could not be revealed by current HLA-II multimer flow cytometry, suggesting the peptide-responsive TCRs may bind their ligand at extremely low affinity. Indeed, binding of soluble GD.C112 TCR to cognate pHLA-II could also not be detected by surface plasmon resonance (data not shown).

### Structure of the 20-mer HLA-DR1 5T4_111–130_ epitope demonstrates extended PFR conformations

**Figure 3.**
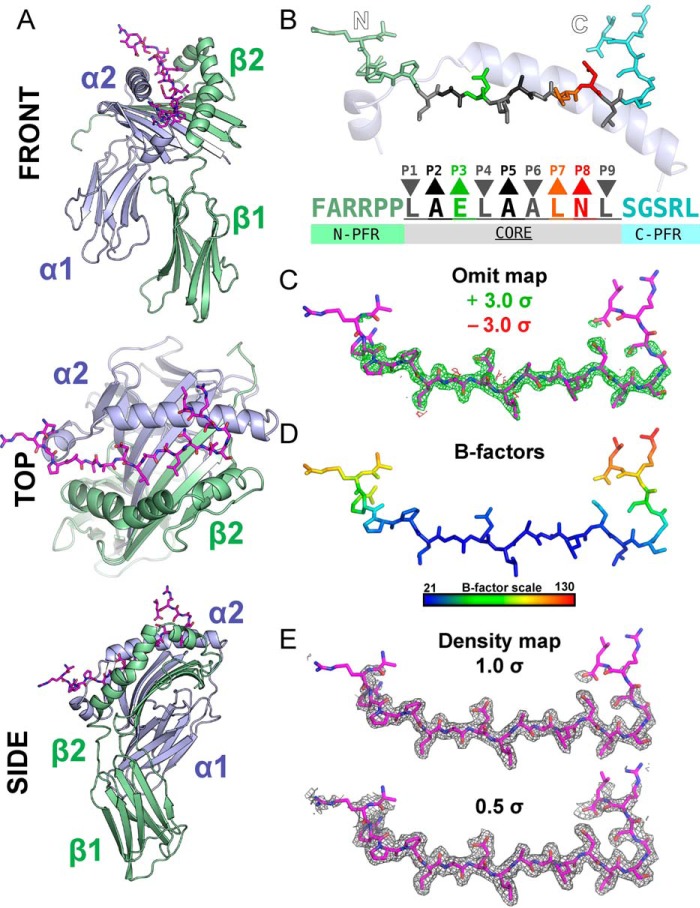
**Structural characterization of the 5T4_111–130_ epitope presented by HLA-DR1.** *A, cartoon* representation of HLA-DR1 (DRα, *blue*; DR1β, *green*) presenting 5T4_111–130_ (*sticks*, *magenta*). *B,* assignment of the 5T4_111–130_ core epitope. HLA-DR1 anchor residues (*gray*; *downward arrowheads* P1, -4, -6, and -9) and peptide flanking residues (N-PFR, *light green sticks*; C-PFR, *cyan sticks*). Potential high enthalpy TCR contact residues are highlighted (P3, *green*; P7, *orange*; P8, *red*). DR1α helix = *blue cartoon. C,* omit map analysis of difference map peaks in the absence of peptide during refinement. Positive difference map peaks (*green mesh*; +3.0 σ) and not negative peaks (*red mesh*; −3.0 σ) exhibit highly related density to refined peptide model (*magenta sticks*; atoms colored: C, *magenta;* O, *red*; N, *blue*). *D,* B-factor analysis indicating a stable core binding region (B-factor <40) flanked by stability extending to both N-terminal and C-terminal flanking regions. Extremities of termini exhibited high flexibility (B-factors >80). *E,* modeled 5T4_111–130_ within refined electron density map contoured at 1.0 σ and 0.5 σ (*gray mesh*).

To gain mechanistic insight into the observed reactivity to tumor-derived antigen, we next sought to investigate the presentation of these epitopes structurally. Generation of TCR–pHLA-II co-complex crystals for the three 5T4 epitope-TCR systems proved unsuccessful, likely due to low receptor-ligand affinity. We did, however, determine the crystal structure of HLA-DR1 in complex with the full-length 5T4_111–130_ 20-mer peptide at 1.95 Å resolution (Table 1 and Fig. 3*A*). 5T4_111–130_ bound via a near-typical HLA-DR1 binding motif incorporating Leu-1, Leu-4, Ala-6, and Leu-9 at P1, P4, P6, and P9 as anchor residues, respectively (Fig. 3*B*). This register was the most probable core-binding region predicted by NetMHCIIpan (26) (Fig. S3*A*). In addition, unbiased omit map analysis provided no evidence of partial occupancy that may result due to register shifting of the epitope (Fig. 3*C*). This therefore defined the core nonamer-binding region of the immunogenic peptide as 5T4_117–125_ (FARRPPLAELAALNLSGSRL; underlined) flanked by six N-terminal PFRs, henceforth N-PFRs (FARRPP) and five C-terminal PFRs, henceforth C-PFRs (SGSRL). Deterioration of electron density at the N and C termini caused an increase in assigned isotropic displacement B-factors within the three most N-terminal residues (**FAR**RPP-**)** and two most C-terminal residues (-SGS**RL**), suggesting that these PFR regions exhibit high flexibility (Fig. 3*D*). As a result, the N-terminal Phe-(−6) residue could not be modeled due to a lack of discernible continuous electron density (Fig. 3*E*).

**Table 1:** **Data reduction and refinement statistics of HLA-DR1–5T4_111–130_**

	HLA-DR1–5T4_111–130_
**Dataset statistics**	
Space group	P 1 2_1_ 1
Unit cell parameters	*a* = 56.96, *b* = 121.29, *c* = 68.96
	α = 90.0°, β = 107.3°, γ = 90.0°
Radiation source	DLS I02*^a^*
Wavelength	0.9795 Å
Resolution range	60.65–1.95 (2.00–1.95) Å
CC-half	0.995 (0.589)*^b^*
Total reflections	239,331 (17,960)
Unique reflections	64,792 (4,807)
Completeness	99.6 (99.8) %
Multiplicity	3.7
*I*/σ	8.3 (1.3)
*R*_merge_ (%)	10.0 (91.3)
Wilson B-factor (A^2^)	31.2
**Refinement statistics**	
Phase determination	Molecular replacement (PHASER)
No. of reflections used	64,762
No. of reflections in *R*_free_ set	3,165
*R*_cryst_ (%)	19.31
*R*_free_ (%)	24.11
Asymmetric unit (ASU) parameters	
Number of copies in ASU	2
Number of non-H atoms	6,784
Deviation from ideal geometry (RMSD)	
Bond lengths	0.0191
Bond angles	1.8659
Chiral volume	0.1086
Ramachandran statistics (MolProbity)	
Most favored	756 (98.1%)
Allowed	15 (1.9%)
Outliers	0 (0.0%)
MolProbity clash score	2.42
MolProbity score	1.48

*^a^* DLS, Diamond Light Source.

*^b^* Values in parentheses represent statistical value for data in the outermost resolution shell.

### PFRs can contribute to HLA anchoring

**Figure 4.**
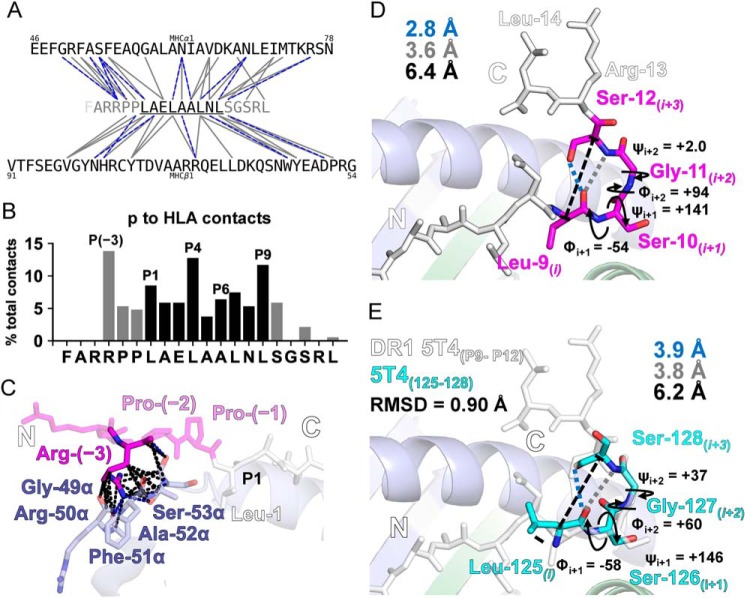
**Lack of HLA influence allows PFR formations reminiscent of native antigen secondary structure.** *A,* peptide to HLA contact network map of van der Waals contacts (≤4.0 Å, *gray lines*) and H-bonds (≤3.4 Å, *blue dashed lines*) between the MHCα1 and MHCβ1 residues and the 5T4_111–130_ peptide (core, *black*; PFRs, *gray*). *B,* peptide to HLA binding contact contribution by each peptide residue. Arg-(−3) contributed the most % contacts throughout the 5T4_111–130_ peptide. *C,* stick representation of the extensive contacts between Arg-(−3) within the N-PFR (*pink*) and -DRα residues 49–53 (*blue*). *D,* geometric parameters of the C-PFR hairpin loop about Leu^9^–Ser^12^ (*pink sticks*). Leu-9*_(i)_* and Ser-12_*(i*+*3)*_ Cα atoms are distanced at 6.4 Å (black *dashed line*). Leu-9*_(i)_* carbonyl hydrogen bound the Ser-12_*(i*+*3)*_ side chain hydroxyl and bound Ser-12_*(i*+*3)*_ amide (*gray dashed line*). Dihedral angles Φ/ψ about Ser-10_*(i*+*1)*_ and Gly-11_*(i*+*2)*_ conform to type II β-hairpin average values: Φ_i+1_ = −60°, ψ_i+1_ = +120°, Φ_i+2_ = +80°, ψ _i+2_ = 0°. *E,* equivalent residues of 5T4 (Leu^125^–Ser^128^) in the published structure of whole 5T4 protein (*cyan sticks*; PDB 4CNM) aligned and overlaid onto residues in-HLA (5T4_111–130_ peptide, *white sticks*). Geometric parameters described in *D* exhibit close similarity.

Crystallization of HLA-DR1–5T4_111–130_ with the full-length unlinked 20-mer peptide represented an opportunity to analyze the role of PFRs in HLA-II presentation and the generation of the functional epitope. Interestingly, we observed contrasting features at each terminus, whereby the N-PFR largely nestled along the -DRα chain, whereas the C-PFR elevated away from the HLA. Such features were accompanied by a differing degree of contacts by N- and C-PFRs to the HLA (Fig. 4*A*). Of the 11 PFRs, five residues contributed to peptide binding to HLA-DR1: N-PFR Arg-(−3), Pro-(−2), and Pro-(−1), as well as the C-PFR Ser-10, Ser-12, and Leu-14 (FAR**RPP**LAELAALNL**S**G**S**R**L**; highlighted in bold, core peptide underlined).

Enumeration of such contacts, however, showed a dramatic drop-off in contacts past Arg-(−3) and Ser-10 within the N- and C-PFRs, respectively (Table 2 and Fig. 4*B*). N-PFRs made nearly 3-fold more contacts with the HLA compared with the C-PFRs. This was mediated by two consecutive prolines (Pro-(−1) and Pro-(−2)), which incorporated a di-proline kink that positioned the N-PFR adjacent to the α2 domain of the peptide-binding groove (Fig. S4). Interestingly, despite being outside the core-binding region, Arg-(−3) displayed the largest number of inter-molecular contacts out of any other peptide residue by contacting five consecutive -DRα residues: Gly-49α–Ser-53α (Table S1). These included 23 van der Waals contacts between the Arg-(−3) peptide backbone (3 contacts) and side chain (20 contacts) atoms (Fig. 4*C*). Moreover, Arg-(−3) was engaged in three short distance (2.75–3.0 Å) hydrogen bonds with -DRα, two from the side chain amine (-NH_2_) of Arg-(−3). Despite the number of contacts made by PFRs to the HLA molecule, we later show that loss or substitution of either N- or C-PFRs did not impact markedly on peptide binding.

**Table 2:** **Contribution of 5T4_111–130_ peptide residues to HLA binding**

Residue	Register	Peptide to HLA contacts		
		vdW*^a^*	H-bonds*^b^*	Total
**N-terminal peptide-flanking residues**				
Arg-(−3)	P(−3)	23	3	26
Pro-(−2)	P(−2)	9	1	10
Pro-(−1)	P(−1)	8	1	9
**Peptide-binding core residues**				
Leu-1	P1	15	1	16
Ala-2	P2	9	2	11
Glu-3	P3	11	0	11
Leu-4	P4	21	3	24
Ala-5	P5	5	2	7
Ala-6	P6	11	1	12
Leu-7	P7	13	1	14
Asn-8	P8	9	1	10
Leu-9	P9	21	1	22
**C-terminal peptide-flanking residues**				
Ser-10	P10	9	2	11
Ser-12	P12	4	0	4
Leu-20	P20	1	0	1
**Totals**				
No. of contacts with PFRs		54	7	61
N-PFRs		40	5	45
C-PFRs		14	2	16
No. of contacts with core		115	12	127
No. of total contacts		169	19	188

*^a^* vdW, van der Waals (≤4.0 Å cut-off).

*^b^* H-bond, hydrogen bonds (≤3.4 Å cut-off).

### Lack of HLA influence on PFRs allows secondary structure reminiscent of native whole antigen

Contacts to the HLA by C-PFRs beyond Ser-10 (P10) were few within the crystal structure. Instead, the C-PFRs formed a hairpin loop structure mediated primarily by intramolecular (peptide to peptide) contacts; causing the protrusion of the peptide away from the HLA binding groove's influence and back to the core peptide region (Fig. 4*D*). This secondary structure was not stabilized by any artifactual crystal contacts (Fig. S5). The hairpin loop formed as a result of four residues, Leu-9, Ser-10, Gly-11, and Ser-12 (assigned *i*, *i*+*1*, *i*+*2*, and *i*+*3,* respectively), forming a tight reverse turn in the C-terminal flank of 5T4_111–130_. The serine/threonine (ST) character loop (27) was stabilized via the Leu-9*_(i)_* backbone carbonyl forming a hydrogen bond to the Ser-12_*(i*+*3)*_ side chain hydroxyl (2.8 Å) and an additional long-range polar interaction (3.6 Å) to the Ser-12_*(i*+*3)*_ amide. Dihedral angles about Ser-10_*(i*+*1)*_ and Gly-11_*(i*+*2)*_ conformed to typical average values observed in type II β-hairpin geometry (28). As a result, Leu-9*_(i)_* and Ser-12_*(i*+*3)*_ Cα atoms were positioned within 7 Å.

Interestingly, examination of the native 5T4 whole protein structure (PDB accession code 4CNM) (29) revealed that the equivalent residues in the native 5T4 whole protein antigen (Leu^125^–Ser^126^–Gly^127^–Ser^128^) also form an ST loop with type II β-hairpin dihedrals (Fig. 4*E*). Structural alignment of the L-S-G-S sequence “in whole protein” and “in HLA” revealed structural similarity about the loop (root mean square deviation 5T4_125–128_ (PDB 4CNM) *versus* HLA-DR1–5T4_125–128_ = 0.90 Å). Thus, we conclude that PFRs can form ordered secondary structural features, reminiscent of native antigen, allowed by a lack of HLA influence on extended PFRs.

### Highly focused specificity to the 5T4_111–130_ core epitope

Having observed interesting structural features within the PFRs we next sought to decipher their importance alongside core residues to forming the functional 5T4_111–130_ epitope using truncated/substituted peptides. Given that PFRs (particularly the N-PFRs) contributed to pHLA stabilization in the crystal structure, we assessed the contribution of residues (both core and PFRs) in HLA-DR1–binding assays. Similarly, the effect of these peptides on cognate T-cell clone activation (GD.D104 IFN-γ ELISA) was assessed given the elevation of C-PFRs toward potential TCR contact.

**Figure 5.**
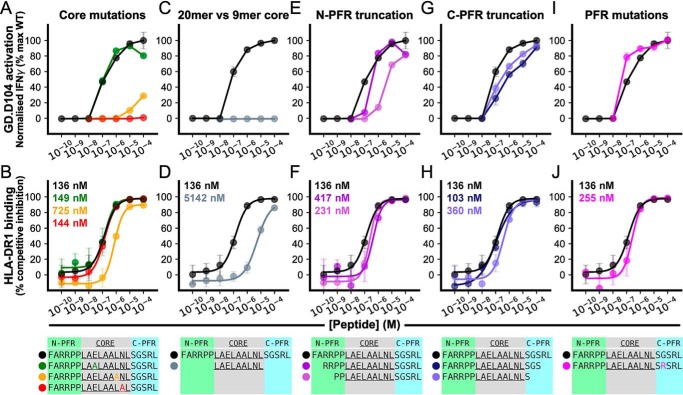
**Highly focused hot spot binding by GD.D104 is enabled by PFRs.** *A,* effect of alanine-substituted 5T4_111–130_ peptides on GD.D104 IFN-γ indicated activation by ELISA. Substitution of Leu-7 and Asn-8 highlight a hot spot of reactivity by GD.D104 within the core-binding region. Peptide colors coded as indicated (*inset table*). IFN-γ response normalized to maximum WT 20-mer response. Representative examples (mean ± S.D.) of two independent experiments performed in at least duplicate is shown. *B,* effect of alanine-substituted 5T4_111–130_ peptides on HLA-DR1 binding in *in vitro* competition assays. Representative examples (mean ± S.D.) of two independent experiments performed in triplicate is shown. *C,* no IFN-γ-indicated activation by GD.D104 to the nonamer core peptide highlighting a requirement for PFRs. *D,* reduction in core 9-mer HLA binding compared with 20-mer. *E,* reduction in activation as a result of N-PFR truncation. *F,* N-PFR truncation resulted in small reductions in HLA-DR1 binding. *G,* reduction in activation due to C-PFR truncation. *H,* minimal effect of C-PFR on HLA-DR1 binding. *I,* modification (Gly^11^ → Arg^11^) of C-PFR hairpin loop resulted in marginally increased activation and maintenance of clone specificity. *J,* minimal effect of PFR mutations on HLA-DR1 binding.

Within the 5T4_111–130_ core, three potential TCR contact residues (Glu-3, Leu-7, and Asn-8) were individually substituted to alanines. Despite the electrostatic and salt-bridge capacity of Glu-3, alanine substitution had no effect on activation, however, alanine substitution of Leu-7 and Asn-8 effectively prevented reactivity of clone GD.D104 (Fig. 5*A*); suggesting specificity of the TCR for long and polar side chain stereochemistry. Each core mutation was not predicted to generate alternate more probable binding registers than the WT peptide (Fig. S3*B*). Alanine substitution had minimal (P7 Leu → Ala) or no (P8 Asn → Ala) effect on peptide binding to HLA-DR1 (Fig. 5*B*), demonstrating that the observed loss of GD.D104 function is due to focused and specific binding of the TCR to a hot spot within the 5T4_111–130_ core binding nonamer.

### PFR residues of 5T4_111–130_ are required for maximal cognate clone activation

Given the focused sensitivity of the TCR to the core bound nonamer (LAELAALNL), it was surprising that clone GD.D104 exhibited no T-cell activation in response to overnight stimulation with the core nonamer peptide, even at extremely high doses (10^−4^ m) of peptide (Fig. 5*C*). Although the core nonamer demonstrated weaker binding to the HLA molecule, as might be expected (Fig. 5*D*), other T-cell clones had previously exhibited detectable T-cell activation to peptides with even weaker HLA-DR1 binding ability (Fig. 2*B*). This suggested that the PFRs played a role in facilitating TCR interactions over and above some enhancement of peptide binding to HLA.

Sequential truncation of the N-PFRs resulted in a stepwise reduction in GD.D104 activation, with N-PFR truncation (FARRPP → PP) requiring high doses of peptide to gain T-cell activation (∼10^−5^ m) (Fig. 5*E*). Even removal of distal residues, at P(−5) and P(−6) caused a reduction of T-cell activation. These residues did not contribute to HLA binding (Fig. 4*A*) nor is there evidence of TCR contacts to these positions in other systems (30). The effect of C-PFR truncations was not as marked, however, truncations still reduced T-cell activation. This was demonstrated by requiring a 10–100–fold increase in peptide concentrations to achieve similar levels of T-cell activation (Fig. 5*G*). Reduction in clone activation was not as a result of a significant reduction in peptide binding to HLA, as both N- and C-PFR truncations resulted in similar HLA binding (Fig. 5, *F* and *H*).

As the TCR appears more focused toward the C-terminal half of the core peptide (P7 and P8) (Fig. 5*A*), reduction of T-cell activation by C-PFR truncation may reflect a disruption to TCR sensing of neighboring C-PFR structural composition, *i.e.* the C-PFR β-hairpin loop. Alteration of the loop by substituting the P11 Gly → Arg (a nonconservative switch that would likely disrupt the C-PFR hairpin loop by restricting dihedral angles about P11) actually increased T-cell activation (clone sensitivity was increased ∼30% of maximal response at 10^−7^ m peptide compared with WT) (Fig. 5*I*). This increase could not be attributed to HLA binding (Fig. 5*J*) or change in predicted core binding frame (Fig. S3*B*).

### PFRs explore the HLA proximity space in molecular dynamics simulations

The above results demonstrate that T-cells specific for three different epitopes derived from the cancer antigen 5T4 express TCRs with low affinity for the pHLA-DR1. A detailed analysis of clone GD.D104 activation by HLA-DR1–5T4_111–130_ revealed a reliance on the PFRs for activation, yet the presence of a distinct secondary structure within the C-terminal PFR (a β–hairpin loop) appeared redundant for T-cell activation. It is therefore not clear as to how this TCR utilizes PFRs for binding and clone activation.

To attempt to answer this question, we conducted all-atom molecular dynamic (MD) simulations at the nanosecond-microsecond time scale in explicit solvent, hypothesizing that PFR mobility not revealed by the static crystallographic data may contribute to PFR tuning of HLA-DR1–5T4_111–130_ recognition. Ten independent simulations of 0.2 μs each were performed using the latest AMBER 16 force field, initiated from both molecules observed in the asymmetric unit.

**Figure 6.**
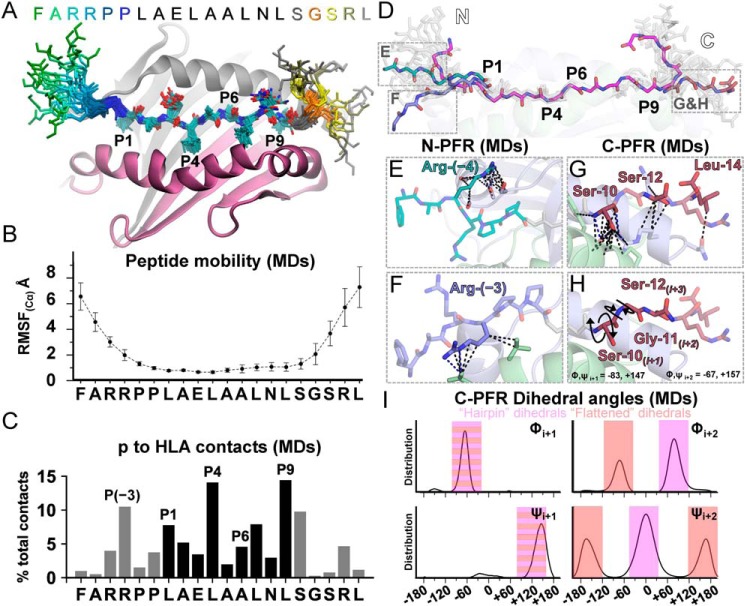
**Conformations explored by PFRs of 5T4_111–130_ in MD simulations.** *A,* representative snapshots from the 20 most populated clusters of the MD trajectories illustrating peptide mobility. Backbone atoms of PFRs are shown as *sticks* and color-coded as indicated (*inset*). Core residues (all atoms) are *blue sticks* with atom coloring as previous. DRα (*gray cartoon*) and DR1β (*purple cartoon*) are represented from the static crystallographic structure. *B,* RMSF of peptide C_α_ atoms. Mean ± S.D. as extracted from 10 independent MD simulations is shown. *C,* percentage of the total number of peptide to HLA contacts (all-atoms including modeled hydrogens = <3.0 Å) calculated from the MDs. *D,* three highlighted alternative PFR conformations represented in MDs. Crystallographic peptide conformation is shown by *pink sticks*. Magnified visualization of *boxed regions* is shown in *E-H* with matched coloring accordingly. *E,* extension of contacts to Arg-(−4) through binding to -DRα residues observed in MD simulations. *F,* alternative conformation whereby Arg-(−3) interacted with -DRβ residues in MD simulations. *G,* representative extended conformation of Ser^10^–Leu^14^ extracted from MD simulations whereby a potential flattening of the C-PFR hairpin loop allows contact with HLA residues in MDs. *H,* this suggested flattened conformation was enabled by inversion of dihedral angles about Gly-11. *I,* distribution of backbone dihedral angles (ϕ, ψ) of residues Ser-10_*(i*+*1)*_ and Gly-11_*(i*+*2)*_ extracted from the MDs. Ideal angles describing the crystallographic hairpin loop and flattened conformation are *boxed* in *pink* and *red*, respectively (±40**°** deviation from ideals).

These MD simulations suggested that both N- and C-PFRs explore a large conformational space (Fig. 6*A*, Video S1). Across the 20-mer peptide, backbone fluctuations exhibited a parabolic distribution (*i.e.* highest at termini) of root mean squared fluctuations (RMSF_Cα_) around the time-averaged positions, highlighting increased peptide mobility due to distance from the core-binding region (Fig. 6*B*). The three most N-terminal (FAR) and C-terminal (SRL) residues exhibited high backbone fluctuations (RMSF_Cα_; 3–7 Å). As expected, residues within the core-binding region remained significantly more rigid (RMSF_Cα_ of 0.7–1.1 Å), however, a degree of rigidity was maintained into the PFR positions proximal to the core residues, with Arg-(−3) (P-3) and Gly-11 (P11) both displaying intermediate RMSF of ∼2.1 Å suggesting maintenance of structural features.

By analyzing the average number of peptide to HLA contacts during the MD simulation time, a similar contact profile compared with the static crystal structure was observed with a slight increase in contacts extending into more distal PFR positions in MD simulations (Fig. 6*C*). Closer examination of representative structures from the most populated clusters extracted from the MD simulations suggested alternative conformations whereby PFRs may stabilize peptide-HLA binding (Fig. 6*D*). In the N-PFR, Arg-(−4) sampled interactions (equating to 4% of total *p-*HLA contacts during the simulation time) with a consecutive pocket of -DRα residues (Gly-49α–Ser-53α); where no contacts were observed in the static crystal structure (Fig. 6*E*). Despite surrounding peptide mobility, Arg-(−3) maintained a considerable contribution (∼10% of total contacts in MDs) by maintaining contact with -DRα residues but also by sampling conformations that allowed binding to -DR1β chain residues Gly-267β and Val-265β (Fig. 6*F*).

Within the C-PFR, we observed high mobility where the C-terminal hairpin loop was maintained in 28% of the MD trajectory as shown by deviation in ϕ, ψ angles at both Ser-10_*(i*+*1)*_ and Gly-11_*(i*+*2)*_ positions (± 40**°** from expected type II β-hairpin geometry). In the remaining simulation time, opening of the hairpin loop was observed allowing contact to the HLA surface (Fig. 6*G*), or potentially transient contacts toward the TCR. This opening was mediated by an inversion of Gly-11_*(i*+*2)*_ dihedrals (Fig. 6*H*). The distribution of the backbone dihedrals of Ser-10_*(i*+*1)*_ and Gly-11_*(i*+*2)*_ in MD simulations indicated that whereas Ser-10_*(i*+*1)*_ exhibits ϕ/ψ values typical of a type II β-turn (ϕ, ψ = −60, 120°), as observed in the static structure, Gly-11_*(i*+*2)*_ could deviate significantly from these expected values (ϕ, ψ = 80, 0°). Instead, typical ϕ, ψ values of unrestricted rotational angles explored by glycine residues was observed (ϕ, ψ = −60°, ±140° within ± 40° deviation) in a significant proportion (12.1%) of trajectory frames (Fig. 6*I*). Thus, the MD simulations suggest that the C-PFR is highly mobile and can readily flip between the hairpin loop and a flattened extended conformation in solution within the submicrosecond time scale. The oscillation between these two orientations may enable this C-PFR to explore the proximal space to facilitate interactions with both the TCR and HLA molecule.

## Discussion

CD4^+^ T-cells have been shown to recognize 5T4-derived antigens in human CRC patients and loss of this recognition has been linked with tumor progression (17). As a result, clonal analysis of CD4^+^ T-cells within the periphery of individuals provides molecular understanding to the initial quality of the T-cell response against 5T4-derived antigens. Moreover, analysis of which 5T4-derived peptides trigger cognate T-cells lends insight into the factors that enable immunogenicity.

We generated CD4^+^ T-cell clones restricted to commonly recognized immunogenic 5T4 peptides presented on HLA-DR1 (20). These clones produced a specific T_H_1 type response to antigen *in vitro*, yet were weakly sensitive compared with virus-reactive clones and exhibited weak/no detectable engagement of cognate ligand by multimer staining or surface plasmon resonance. Our data highlight that, at the clonal level, despite the immunogenicity of these peptides, the quality, *i.e.* sensitivity of the CD4^+^ T-cell response to such epitopes is poor compared with nonself epitopes. This may reflect a thymic selection process on self-antigens that deletes T-cells responding to high affinity peptide ligands (31). Thymic expression of 5T4 has indeed been shown in mouse at the RNA level (32). Adequate peptide to HLA-DR1 binding capacity was observed for two 5T4-derived peptides, although binding was slightly weaker than pathogen-derived peptides such as IAV-HA_306–319_. One 5T4 epitope exhibited weak HLA-DR1 binding capacity that may in part explain the relative poorer sensitivity of this cognate clone compared with the two other 5T4-reactive clones in ELISA and ELISPOT assays. Overall, the contribution of peptide-HLA stability to the selection and sensitivity of anti-tumor CD4^+^ clones should not be overlooked.

To focus our investigation, we were able to solve the structure of one of these epitopes (5T4_111–130_) bound by HLA-DR1 at high resolution, which defined the core binding nonamer. Subsequently, alanine substitution of residues within this core resulted in loss of clone reactivity. These modified peptides, along with all mutated/truncated peptides of 5T4_111–130_ tested, were not predicted to induce new preferential binding motifs. It is therefore likely that loss of clone reactivity was not as a result of epitope frame-shifting; suggesting instead that the generated cognate clone focused binding to a hot spot within the core peptide, where Leu-7 and Asn-8 were essential for immunogenicity.

Despite exhibiting highly focused binding to the epitope core of 5T4_111–130_, the core peptide alone was insufficient to trigger cognate clone activation in *in vitro* assays. This was coupled with a significant decrease in peptide-HLA binding capacity exhibited by the core alone compared with the 20-mer version. Binding, however, still exceeded levels that will trigger T-cells, suggesting a requirement of PFRs for optimal TCR-pHLA interactions.

PFR requirement for immunogenicity is interesting given our structural observations of the 5T4_111–130_ PFRs. We first identified that HLA-DR1 can maintain considerable contact with presented peptides between P3 and P12 positions. Such contacts in the crystal structure suggest that a 15-mer peptide may be optimal for HLA-DR1–5T4_111–130_ complex stability: 3-mer N-PFRs and 3-mer C-PFRs. Using cognate clone activation and HLA-DR1–binding assays, however, we show that although truncation of the epitope outside these limits could affect binding, the effects on T-cell activation were more significant. A general optimal length for HLA-II binding has been previously simulated at 18–20 amino acid length for a dataset of antigenic HLA-II peptide sequences (33). By performing MD simulations, we suggest that the PFRs of epitopes are highly mobile and explore conformations: (i) toward the HLA-potentially extending peptide-HLA contacts further outwards to PFR extremities, and (ii) into space away from the HLA toward potential TCR engagement.

Although the N-PFR made close contact to the HLA, the C-PFR elevated away from the HLA surface volume and into proximity of the peptide core. This elevation formed by a ST loop was enabled through residue-specific peptide to peptide interactions. A similar hairpin turn structure, a type II β-turn mediated by backbone interactions, has been described previously whereby, in this system, hairpin disruption resulted in significant abrogation of cognate clone activation (6). In our 5T4_111–130_ system, despite a reduction in clone sensitivity to C-PFR truncation, abrogation of GD.D104 clone activation was not as drastic as alteration of core binding hot spot residues, to which the C-PFR was spatially located.

Interestingly, the 5T4_111–130_ C-PFRs resembled that of native whole protein antigen. Such refolding of peptide sequences in-HLA is likely due to a loss of influence by the HLA, as shown by diminishing peptide to HLA contacts to extended PFRs. Consequently, inherent peptide folding geometry, due to native whole protein amino acid composition, may no longer be unraveled and pinned down through HLA presentation. Thus, antigen processing may be tuned to create a peptide ligandome, which allows the presentation of peptides that benefit from PFR length enhanced immunogenicity while limiting peptides to a length that is controllable by the boundary of influence, *i.e.* the span of the peptide binding groove of HLA-II molecules.

Given a dependence on PFRs to initiate any GD.D104 response to 5T4_111–130_ it may be reasonably assumed that TCR engagement is sensitive to the structural features of 5T4_111–130_ PFRs. Mutation of a PFR residue designed to drastically alter the PFRs structural composition, however, did not negatively affect clone activation, instead showing a potential increase in immunogenicity. In contrast, even a conservative (Leu → Ala) modification to a core residue of 5T4_111–130_ had little impact on HLA binding but strikingly reduced cognate T-cell clonal activation. Thus, cognate clone reactivity and specificity to 5T4_111–130_ was focused on core residues but enabled through PFRs. This observation supports the notion that PFRs are an amenable target to improve the immunogenicity of HLA-II–restricted T-cells through modifications that enhance TCR binding (4, 13, 34), whereas maintaining ligand specificity. Moreover, relevant neo-antigens may be missed through searching for core-only neo-epitopes without considering the impact mutations within PFRs may have on TCR discrimination of antigens as nonself. Indeed, human thymic HLA-DR peptide repertoires consist of PFR containing peptides, thus PFRs may influence central tolerance (35). Further studies into the impact of PFRs is warranted to decipher whether tumor-epitopes may indeed be more dependent on PFR tuning, compared with nonself systems, due to the weak sensitivity of the 5T4 CD4^+^ T-cell response observed here at the clonal level.

We therefore envisage extended PFRs as highly mobile entities of HLA-II epitopes that make transient, but extremely important, interactions with the HLA and TCR. Such transient interactions may have a summative effect that tunes core-focused engagement by the TCR repertoire. Thus, this study advances our understanding of the role PFRs play during T-cell recognition, extends the definition of what constitutes an HLA-II peptide epitope, and suggests PFRs could be targeted in future therapies. Furthermore, unlike HLA-I where the peptide is generally constrained within the peptide-binding cleft, the open-ended groove of HLA-II enables the presentation of natively folded protein conformations outside of the linear peptide nonamer core. These data demonstrate that TCR recognition of pHLA-II is more complex than previously appreciated, with interesting implications for pHLA-II–restricted TCR antigen specificity during CD4^+^ T-cell–mediated immunity.

## Experimental procedures

### Generation and culture of T-cell clones

CD4^+^ T-cell clones were generated using a T-cell library cloning method outlined previously (22). PBMCs were isolated from an HLA-DR1^+^ donor, enriched for CD4^+^ cells by magnetic microbead separation (Miltenyi Biotec), and expanded using human T-activator CD3/CD28 Dynabeads® (Life Technologies) at a cell to bead ratio of 1:2 for 14 days. Expanded cells were screened for IFN-γ release in response to a pool of 5T4 candidate peptides (1 × 10^−5^ m) via enzyme-linked immunospot assay (ELISpot) using T2-DR1^+^ transduced cells as antigen-presenting cells produced as described previously (22). Peptide responsive lines were pooled, expanded with a 1 × 10^−5^ m individual 5T4 peptide for 4 h before enrichment by IFN-γ isolation (Miltenyi Biotec). The enriched line was tested for reactivity to individual 5T4 peptide via IFN-γ ELISpot before single cell cloning by serial dilution. The DCD10 clone was generated and described previously (34).

Clones were maintained in culture at 3 × 10^6^ cells/ml in 10% fetal calf serum, 2 mm l-glutamine, 100 IU/ml of penicillin, and 100 μg/ml of streptomycin, 0.02 m HEPES, 1 mm nonessential amino acids, 1 mm sodium pyruvate, and 200 IU/ml of human recombinant IL-2 (Proleukin®) media. T-cells were expanded fortnightly using 1 μg/ml of phytohemagglutinin (Alere, Cheshire, UK), in the presence of irradiated (3100 gray) allogeneic PBMC feeder cells from three healthy donors.

### Peptide-sensitivity assays

T-cell peptide activation assays were performed by co-culturing T-cell clones with T2-DR1 cells as APCs in the presence of WT or modified/truncated 5T4 peptides at a 2:1 APC to T-cell ratio. Prior to the assay, T-cell clones were rested in RPMI 1640 media, 5% fetal calf serum, 2 mm l-glutamine, 100 IU/ml of penicillin, and 100 μg/ml of streptomycin overnight. Serially diluted peptides were added to the co-culture and incubated overnight at 37 °C, 5% CO_2_. Culture supernatants were harvested for analysis of human IFN-γ, TNFα, and MIP-1β using enzyme-linked immunosorbent assay (ELISA) according to the manufacturer's protocol (R&D Systems). All ELISA data were background subtracted using baseline cytokine/chemokine response to no peptide controls, analyzed, and presented using *matplotlib* (36).

### Detection of T-cell activation by ELISpot assay

T-cell activation assays were performed by co-culturing peptide-pulsed T2, T2-DR1, or autologous B lymphoblastoid cells as APCs and activation was determined by a IFN-γ release ELISpot assay (Mabtech). 5 × 10^6^ APCs were pulsed at 37 °C, 5% CO_2_ for 2 h with 5T4 peptides (10^−6^-10^−5^ m) before washing twice with PBS. APCs were incubated with 10 μg/ml of anti-DR (L243 clone) antibody or media control for 1 h at 37 °C and washed with PBS. APCs were then plated with 300 rested GD.D104 T-cells onto prepared IFN-γ ELISpot plates, cultured overnight, stained, and developed as per manufacturer's instructions and imaged using an ImmunoSpot® analyzer (Cellular Technology Limited).

### Peptide-binding assays

Ability of peptides to bind HLA-DR1 molecules was determined by detection of competitive binding of candidate peptides to a biotinylated marker peptide (37). 60 μl of peptide exchange reactions were prepared in 20 mm MES, 140 mm NaCl, 0.02% NaN_3_, pH 5 buffer with 0.1 μg of refolded HLA-DR1-CLIP_105–117_ (SKMRMATPLLMQA), 4.5 nm N terminally biotinylated CLIP_99–117_ (bt-LPKPPKPVSKMRMATPLLMQA) marker peptide, and 10-fold serially diluted (10^−4^-10^−10^ m in triplicate) candidate test peptide. Meanwhile, wells of half-area high-bind ELISA plates were incubated with 20 ng/μl of anti-DR capture antibody (L243 clone) in PBS (50 μl). After overnight incubation (ELISA plate at room temperature; peptide exchange at 37 °C), ELISA plates were washed once with PBS, 0.02% Tween (PBS-T), blocked with 3% BSA, PBS, 0.02% NaN_3_ (3% PBS) at room temperature for 1 h, then washed (3× PBS-T, 3× PBS). Peptide exchange reactions were neutralized using 1 m Tris, 10% BSA, 1% Tween, 0.02% NaN_3_, pH 10, solution (10 μl) and transferred (70 μl) to the anti-DR–coated ELISA plate. After 1 h 20 min (room temperature), peptide exchange reactions were discarded, plates were washed (3× PBS-T, 3× PBS), then incubated with streptavidin-HRP (diluted in 3% BSA-PBS solution according to manufacturer's recommended dilution for ELISAs) for 20 min (R&D Systems). After washing (3× PBS-T, 3× PBS), HLA-DR1 bound bt-CLIP marker peptide was detected using HRP substrate (R&D Systems). Candidate test peptide binding was evaluated by the ability to competitively inhibit detected bound bt-CLIP marker compared with bt-CLIP marker only controls (% competitive inhibition). Peptide-binding assays were plotted using *matplotlib* and IC_50_ values were calculated by fitting the four-parameter log(inhibitor) response function using *SciPy* (33). Peptide binding predictions were performed using NetMHCIIpan version 3.2 (26).

### Generation of HLA-DR1 molecules

HLA-DR1 molecules were *in vitro* refolded from inclusion bodies as described previously (38). DRα*0101 (Uniprot: P01903, residues 26–207) and HLA-DRβ1*0101 (Uniprot: P04229, residues 30–219) inclusion bodies were produced in Rosetta^TM^(DE3) competent BL21 strain-derived *Escherichia coli* cells (Novagen). Prepared HLA-DRα and -DR1β chain inclusion bodies in 8 m urea, 20 mm Tris, pH 8.1, 0.5 mm EDTA, pH 8.1, were purified by Hi-Trap Q-Sepharose High Performance anion exchange chromatography using an AKTA Pure FPLC (GE Healthcare Life Sciences).

To refold, 5 mg/liter of each, HLA-DRα and -DR1β inclusion bodies were added to 25% glycerol, 20 mm Tris, 1 mm EDTA, 20 mm NaCl, 1.48 g/liter of cysteamine hydrochloride, and 0.83 g/liter of cystamine hydrochloride supplemented with 0.5 mg/liter of peptide (Peptide Protein Research Ltd.) and stirred vigorously for 1 h before incubation for 72 h at 4 °C. Refold mixture was next buffer exchanged with PBS using 10 kDa MWCO filtration units (Sartorius AG) and concentrated with centrifugal filter units (Merck Millapore).

Conformational HLA-DR1 molecules were purified using an anti-DR (clone L243) antibody affinity purification column produced using a Pierce^TM^ Protein A IgG Plus Orientation Kit (ThermoFisher Scientific) eluting bound HLA-DR1 via 50 mm CAPS, pH 11.5. Samples were further purified into 10 mm Tris, 10 mm NaCl, pH 8.1, via size exclusion chromatography (SEC) using a Superdex 200HR gel SEC column (GE Healthcare Life Sciences).

### HLA-II multimer staining

Biotinylated HLA-DR1 molecules were refolded from inclusion bodies as above with an additional C-terminal biotinylation signal sequence (GLNDIFEAQKIEWHE; AviTag^TM^) ligated to the HLA-DRA*0101 sequence described, via a flexible linker (GSGG). Refolded HLA-DR1 samples were biotinylated using a *BirA* biotinylation kit (Avidity) and incubating overnight at room temperature. Biotinylation efficiency was assayed by a SDS-PAGE streptavidin shift assay (39).

Multimers were assembled on the day of use by combining 2 μl of phycoerythrin-labeled dextramer backbone (Immudex) with 0.5 μg of monomer per stain. T-cells were treated with dasatinib as described previously (23) before staining with multimer for 30 min on ice. Multimer staining was boosted using an anti-phycoerythrin secondary antibody as described previously (24). Cells were then stained for viability using LIVE/DEAD® Fixable Violet Dead Cell Stain (Invitrogen) and surface CD4 expression (αCD4-APC). Stained cells were analyzed on a FACS Canto II (BD Biosciences) and data were evaluated in FlowJo (Tree Star Inc.).

### Protein crystallization, diffraction, and model refinement

HLA-DR1–5T4_111–130_ crystals were grown using sitting drop vapor diffusion and crystal microseed hanging drop vapor diffusion. Initial screening plates were performed using an Art-Robbins Gryphon robot (Art Robbins Instruments, LLC.) by dispensing 200 nl of protein into 200 nl of reservoir candidate screen solution and incubation at 18 °C. Nondiffracting HLA-DR1–5T4_111–130_ crystals were harvested for production of crystal microseeds using a Seed Bead^TM^ Kit (Hampton Research Corp.). HLA-DR1–5T4_111–130_ crystals were grown in 0.02 m sodium/potassium phosphate, 0.1 m Bistris propane, pH 7.5, 20% PEG 3350 solution in the presence of combined HLA-DR1–5T4_111–130_ microseeds grown in conditions detailed in Table S2 and Fig. S6 via manually set drops (1 μl of reservoir solution, 1.5 μl of protein sample, and 0.5 μl of microseed stock).

Cryopreserved crystals were exposed to X-rays and their diffraction recorded at Diamond Light Source (Oxfordshire, UK) at 100 K using a wavelength of 0.9795 Å. Observed reflection intensities were estimated using XIA2 (40), and data were analyzed with AIMLESS and the CCP4 package (41). Phases were obtained with molecular replacement using PHASER (42). Models were refined using graphical manipulation in COOT (43) and computationally refined through iterative TLS and conjugate gradient refinement using REFMAC5 (44) until convergence. Graphical representations were prepared using PyMOL (45). Contact tables were generated using NCONT (CCP4). Reflection data and final model coordinates were deposited to the Protein Data Bank under the code 6HBY.

### Molecular dynamics simulations

Two simulation systems were prepared based on the two molecules resolved in the crystal structure of the HLA-DR1–5T4_111–130_ complex. The unresolved N-terminal residues of HLA-DR1 and 5T4_111–130_ were modeled at extended conformations to avoid steric clashes. Protonation states were calculated for physiologically relevant conditions (pH 7.4 and *I* = 0.15 M) using the H++ server (46). Both systems were solvated into truncated octahedral boxes with TIP3P waters extending up to 12 Å around the solute and the appropriate number of Na^+^ to neutralize the total charge of each system were added. AMBER *ff14SB* force field parameters (47) were applied using the LEaP module of AMBER version 16 (48).

Molecular dynamics simulations were carried out using the GPU-accelerated version of PMEMD (49), employing the equilibration protocol and parameters as previously described (50). Five independent production runs for each system were performed in the isothermal isobaric ensemble at 310 K for 200 ns, yielding an aggregate of 2,000 ns. A subset of 20,000 structures sampled every 0.1 ns were clustered using a hierarchical agglomerative approach with a minimum distance between clusters of 2.5 Å, after mass-weighted, root mean square deviation fitting of the HLA-DR1 Cα atoms using the CPPTRAJ module of AmberTools version 16 (51).

## Author contributions

B. J. M., G. D., A. P., A. G.-W., M. B., B. S., T. E., A. Gallimore, P. R., D. K. C., and A. Godkin formal analysis; B. J. M., G. D., A. P., A. G.-W., A. Gallimore, P. R., and D. K. C. validation; B. J. M., G. D., A. P., A. G.-W., G. H. M., A. S., M. B., A. Gallimore, P. R., D. K. C., and A. Godkin investigation; B. J. M. and A. P. visualization; B. J. M., G. D., A. P., A. G.-W., A. S., M. B., A. K. S., A. Gallimore, P. R., and D. K. C. methodology; B. J. M., A. P., P. R., and D. K. C. writing-original draft; B. J. M., A. P., A. G.-W., G. H. M., T. E., A. K. S., A. Gallimore, P. R., and D. K. C. writing-review and editing; A. P. software; M. B. and B. S. data curation; B. S., T. E., A. K. S., A. Gallimore, P. R., and D. K. C. resources; T. E., A. K. S., A. Gallimore, P. R., D. K. C., and A. Godkin conceptualization; A. K. S., A. Gallimore, P. R., and D. K. C. funding acquisition; P. R., D. K. C., and A. Godkin supervision; P. R. and D. K. C. project administration.

## Supplementary Material

Supporting Information

## Footnotes

5 The abbreviations used are:

TCR: T cell receptor
HLA: human leukocyte antigen
(p)HLA-II: (peptide) human leukocyte antigen class II
PFR(s): peptide flanking residue(s)
N-PFRs: N-terminal PFRs
C-PFRs: C-terminal PFRs
APCs: antigen presenting cells
IAV: influenza A virus
HA: haemagglutinin
HLA-DR1: HLA-DRα1*0101, β1*0101
ELISpot: enzyme-linked immunospot
PDB: Protein Data Bank
MD: molecular dynamic
RMSF: root mean squared fluctuation
CRC: colorectal cancer
ST loop: serine-threonine loop
PBMCs: peripheral blood mononuclear cells
CLIP: class II-associated invariant chain peptide
MHC: major histocompatibility complex
CAPS: 3-(cyclohexylamino)-1-propanesulfonic acid
Bistris propane: 1,3-bis[tris(hydroxymethyl)methylamino]propane
IFN: interferon
TNF: tumor necrosis factor
HRP: horseradish peroxidase.
