# Supplementary material for: Human leukocyte antigen (HLA) class II peptide flanking residues tune the immunogenicity of a human tumor-derived epitope
Source: J Biol Chem. 2019 Oct 16;294(52):20246–58. doi: 10.1074/jbc.RA119.009437 (PMC6937582; doi:10.1074/jbc.RA119.009437)
Supplement: Supporting Information [file supp_294_52_20246__index.html]

Human leukocyte antigen (HLA) class II peptide flanking residues tune the immunogenicity of a human tumor-derived epitope — PFRs influence CD4+ T-cell response to 5T4 — Supporting Information 

# Human leukocyte antigen (HLA) class II peptide flanking residues tune the immunogenicity of a human tumor-derived epitope

## Supporting Information

- Supporting Information revision 3 - Revised Supporting Information in full-formatted PDF in response to reviewers comments.
- Supporting Video S1 - Supporting video describing results of molecular dynamics simulations. Title: MD trajectories illustrating peptide mobility.
