# Supplementary material for: Human leukocyte antigen (HLA) class II peptide flanking residues tune the immunogenicity of a human tumor-derived epitope
Source: J Biol Chem. 2019 Oct 16;294(52):20246–58. doi: 10.1074/jbc.RA119.009437 (PMC6937582; doi:10.1074/jbc.RA119.009437)
Supplement: Supporting Information [file supp_RA119.009437_152962_1_supp_406004_pzb39x.pdf]

Human leukocyte antigen (HLA) class II peptide flanking residues tune the immunogenicity of a human tumor-derived epitope

Bruce J. MacLachlan[a,†], Garry Dolton[a], Athanasios Papakyriakou[b,c], Alexander Greenshields-Watson[a], Georgina H. Mason[a], Andrea Schauenburg[a], Matthieu Besneux[a], Barbara Szomolay[a], Tim Elliott[c,d], Andrew K. Sewell[a], Awen Gallimore[a], Pierre Rizkallah[a], David K. Cole[a,#,*], Andrew Godkin[a,f,*,&]

From the [a]Division of Infection & Immunity and Systems Immunity Research Institute, Cardiff University, CF14 4XN, UK; [b]Centre for Biological Sciences, Faculty of Natural & Environmental Sciences, University of Southampton, SO17 1BJ, UK; [c]Institute for Life Sciences, University of Southampton, Southampton, SO17 1BJ, UK; [d]Centre for Cancer Immunology, University of Southampton, Faculty of Medicine, University Hospital Southampton, SO16 6YD, UK [f]Department of Gastroenterology and Hepatology, University Hospital of Wales, CF14 4XN, Cardiff, UK.

Running title: *PFRs influence CD4$^+$ T cell responses to 5T4*

[†] Present address: Monash Biomedicine Discovery Institute, 19 Innovation Walk, Clayton, Victoria, 3800, Australia
[#] Present address: Immunocore Ltd, Milton Park, Abingdon, Oxfordshire, OX14 4RX, UK

*These authors contributed equally to the study

[&]To whom correspondence should be addressed: Andrew Godkin; Division of Infection & Immunity and Systems Immunity Research Institute, Cardiff University, Henry Wellcome Building, University Hospital of Wales, Heath Park, CF14 4XN, UK; GodkinAJ@cardiff.ac.uk; Tel.+44 (0)29 2068 7003

Supporting Information:

Table S1 Intermolecular contacts between Arg-(−3) and HLA-DR1 .... **S-2**

Table S2. Crystallization conditions from which HLA-DR1-5T4$_{111-130}$ dataset was obtained........... **S-3**

Figure S1. Specific clone reactivity to cognate 5T4 peptide **S-4**

Figure S2. Flow cytometric analysis of CD4 and TCR expression of the three 5T4-reactive T-cell clones prior to multimer staining **S-5**

Figure S3. Core binding register predictions of 5T4$_{111-130}$ and mutated/truncated peptides................**S-6**

Figure S4. Additional intermolecular contacts between 5T4$_{111-130}$ PFRs and HLA-DR1 .... **S-7**

Figure S5. Crystal packing and symmetry interactions of the HLA-DR1 5T4$_{111-130}$ structure **S-8**

Figure S6. Optimized growth of HLA-DR1-5T4$_{111-130}$ protein crystals through microseeding **S-9**

Video S1. MD trajectories illustrating peptide mobility. **S-10**

Table S1 Intermolecular contacts between Arg-(−3) and HLA-DR1

Peptide		MHC			Contact	
**Residue**	**Atom**	**Chain**	**Residue**	**Atom**	**Distance**	**Type**
Peptide backbone contacts						
**Arg-(−3)**	C	DRα	Ser-53	Oγ	3.84	VW
	O	DRα	Ser-53	N	3.78	VW
	O	DRα	Ser-53	Cβ	3.67	VW
	O	DRα	Ser-53	Oγ	2.82	HB
Peptide side chain contacts						
**Arg-(−3)**	Cβ	DRα	Ala-52	Cα	3.84	VW
	Cβ	DRα	Phe-51	C	3.83	VW
	Cβ	DRα	Phe-51	O	3.67	VW
	Cβ	DRα	Ala-52	N	3.8	VW
	Cβ	DRα	Ala-52	C	3.73	VW
	Cβ	DRα	Ser-53	N	3.93	VW
	Cγ	DRα	Arg-50	C	3.96	VW
	Cγ	DRα	Arg-50	O	3.43	VW
	Cδ	DRα	Arg-50	C	3.97	VW
	Cδ	DRα	Arg-50	O	3.71	VW
	Cδ	DRα	Gly-49	O	3.3	VW
	Nε	DRα	Gly-49	O	3.85	VW
	Cζ	DRα	Gly-49	O	3.67	VW
	Cζ	DRα	Ala-52	O	3.93	VW
	Cζ	DRα	Ser-53	Cβ	3.89	VW
	NH1	DRα	Ser-53	Cβ	3.58	VW
	NH2	DRα	Ala-52	C	3.8	VW
	NH2	DRα	Ser-53	Cα	3.96	VW
	NH2	DRα	Gly-49	C	3.81	VW
	NH2	DRα	Gly-49	O	2.75	HB
	NH2	DRα	Ala-52	O	2.99	HB
	NH2	DRα	Ser-53	Cβ	3.91	VW
**No. of total contacts**						**26**
vdW						23
H-bonds						3
**No. of contacts by Arg-(−3) backbone**						**4**
vdW						3
H-bonds						1
**No. of contacts by Arg-(−3) side chain**						**22**
vdW						20
H-bonds						2

vdW = van der Waals (=< 4.0 Å cut-off), H-bonds = hydrogen bonds (=< 3.4 Å cut-off).

Table S2 Crystallization conditions from which HLA-DR1-5T4$_{111\text{-}130}$ dataset was obtained

	**HLA-DR1-5T4$_{111\text{-}130}$**
**Crystallisation conditions**	
**Primary screening**	
Screen conditions	
TOPS D1	0.1 M Hepes pH 7.0, 15 % Glycerol, 15 % PEG 4000
TOPS F12	0.1 M Tris pH 7.5, 0.2 M Amonium Sulphate, 25 % PEG 8000
PACT G10	0.02 M Sodium/potassium phosphate, 0.1 M Bis-Tris propane pH 7.5, 20 % PEG 3350
Protein concentration	4 mg/mL
Total drop volume	200 nL
**Seeding**	
Parent seed protein	Self (HLA-DR1 5T4$_{111\text{-}130}$)
Parent seed screen conditions	TOPS D1, TOPS F12, PACT G10
Dilution volume	1 in 100 (5 mL)
Dilution buffer	PACT G10
Reservoir buffer	PACT G10
Protein concentration	4 mg/mL
Total drop volume	3 µL

TOPS = TCR-pMHC Optimised Protein Screen (47), PACT = PACT premier™ HT-96 MD1-36 (Molecular Dimensions), % = % w/v.

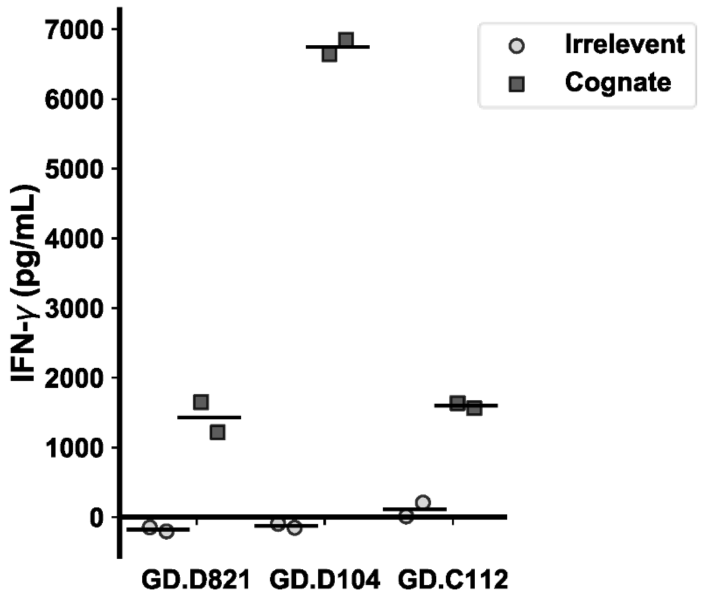

Figure S1. Specific clone reactivity to cognate 5T4 peptide. GD.D821, GD.D104 and GD.C112 IFN-γ release in response to stimulation with 10^{-5} M irrelevant or cognate peptides as measured by ELISA. Each clone was cross-stimulated with the two non-cognate 5T4 peptides recognized by the three clones as irrelevant peptides controls i.e. the $5T4_{11\text{-}30}$ restricted GD.D821 was stimulated with $5T4_{111\text{-}130}$ and $5T4_{371\text{-}390}$ and *vice versa*. Scatter points = individual response to each irrelevant peptide, horizontal line = mean response to both irrelevant peptides.

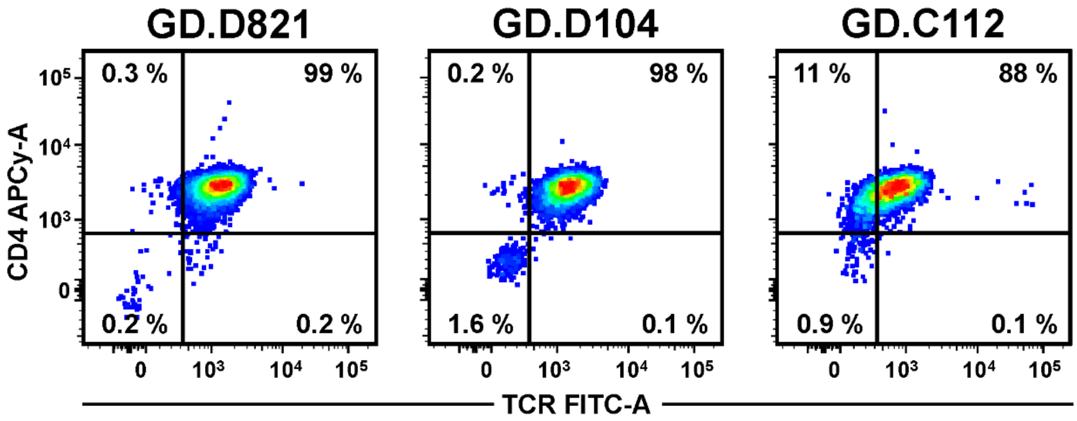

Figure S2. Flow cytometric analysis of CD4 and TCR expression of the three 5T4-reactive T-cell clones prior to multimer staining. Each clone exhibited expression of both antigen receptor and co-receptor as indicated by > 88 % of cells CD4+ TCR+. Quadrant gates set according to FMO/unstained negative control populations. Representative of two independent experiments.

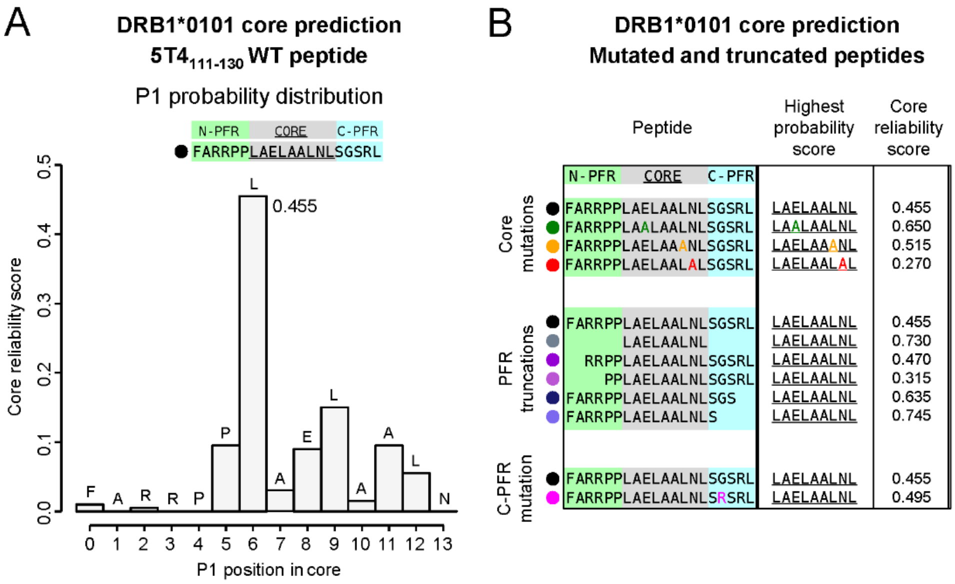

Figure S3. Core binding register predictions of 5T4$_{111\text{-}130}$ and mutated/truncated peptides. (*A*) Predicted core binding region of wild type 5T4$_{111\text{-}130}$ performed by NetMHCIIpan v3.2 (FARRPPLAELAALNLSGSRL; core underlined). Probability distribution of predicted residue located at the P1 position of HLA-DRB1*0101 (DR1), highlighting the most probable core register incorporates Leu (5T4$_{117}$) at position P1 as observed in the crystal structure. (*B*) Predicted core binding regions of mutated/truncated peptides of 5T4$_{111\text{-}130}$ used in HLA binding/T-cell clone activation assays. NetMHCIIpan predicts all peptides most likely retain the same core register as wild type 5T4$_{111\text{-}130}$.

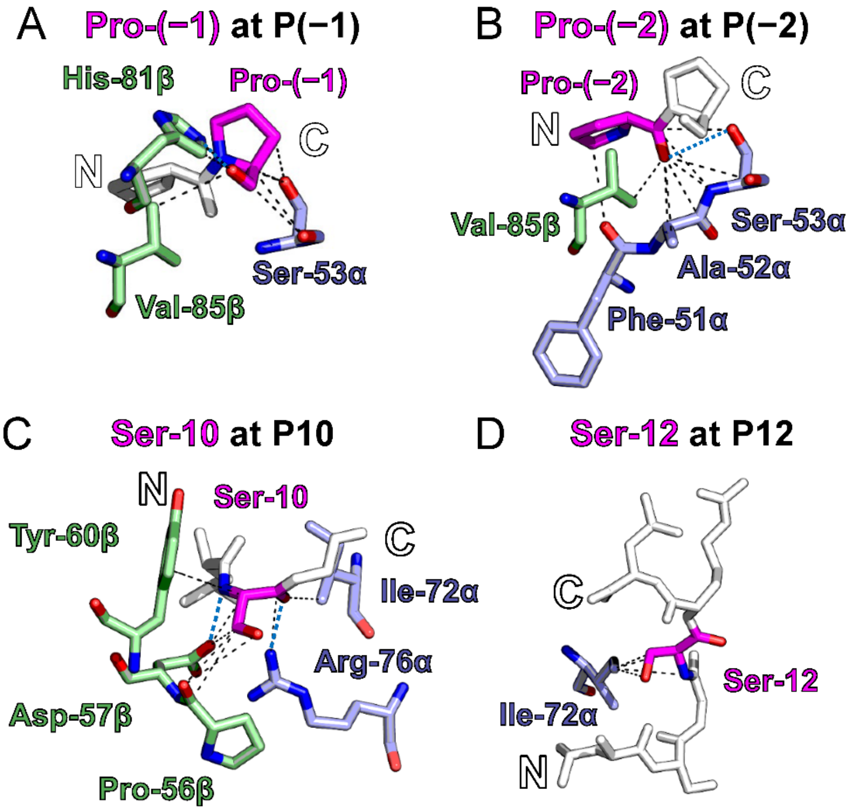

Figure S4. Additional intermolecular contacts between 5T4$_{111\text{-}130}$ PFRs and HLA-DR1. (*A*) Pro-(−1) (magenta) interacting with Ser-53α, His-81β and Val-85β. Van der Waals contacts (=< 4.0 Å; black dashed lines), H-bonds (=< 3.4 Å; blue dashed lines), -DRα contacting residues (blue), -DR1β contacting residues (green). (*B*) Pro-(−2) interacting with Phe-51α, Ala-52α & Ser-53α and Val-85β. Together, Pro-(−1) and Pro-(−2) form a diproline kink. (*C*) Ser-10 interacting with DR1α residues Ile-72α and Arg-76α and DR1β residues Pro-56β, Asp-57β and Tyr-60β. (*D*) Ser-12 interacting with DR1α residue Ile-72α. Minimal contacts were observed including no side chain hydrogen bonding between the Ser-12 hydroxyl group and the HLA molecule.

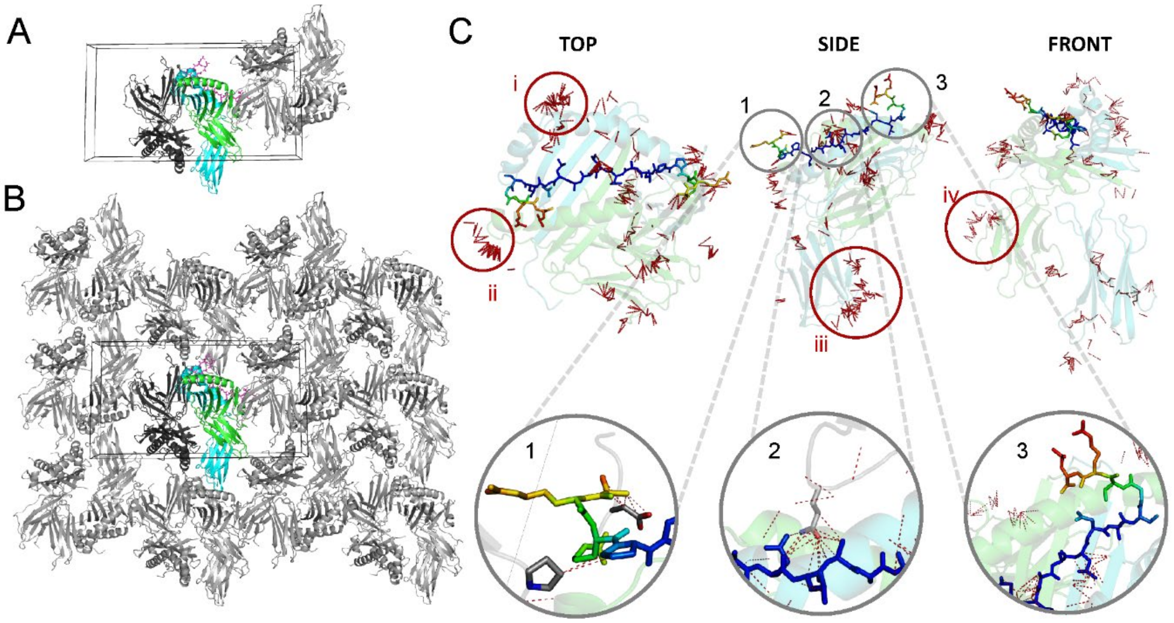

Figure S5. Crystal packing and symmetry interactions of the HLA-DR1 $5T4_{111\text{-}130}$ structure. (*A*) Cartoon and stick representation of HLA-DR1 $5T4_{111\text{-}130}$ within the unit cell (black box) of the analyzed crystal structure. The asymmetric unit consisted of two HLA-DR1 $5T4_{111\text{-}130}$ copies: chain A, B & C (green, cyan & pink respectively) from which crystallographic analyses are calculated, and a second copy D, E & F (dark grey). Symmetry mate within the unit cell is also shown (light grey). (*B*) Cartoon representation of HLA-DR1 $5T4_{111\text{-}130}$ within the observed crystal lattice showing symmetry mates within a single two-dimensional lattice (light grey). Asymmetric unit colored as in Fig. S4. *A*. (*C*) Cartoon and stick representation of crystal packing interactions. All theoretical crystal contacts (distance ≤ 4.0 Å) between the HLA-DR1 $5T4_{111\text{-}130}$ (chain A, B & C of asymmetric unit) to the second copy of HLA-DR1 $5T4_{111\text{-}130}$ within the asymmetric unit and contacting symmetry mates. Contacts are shown as red dashed lines. Contact hotspots (red circles) included the β1 helix center (i), the α1 helix C-terminal edge (ii), β2 domain back (iii) and α2 domain side (iv). Some contacts by Pro-2 and Arg-4, to flexible loop regions of symmetry mates, was observed within the peptide N-terminus (grey circle 1). In addition, DR1β-Glu-110, within a flexible loop of the DR1 β2 domain was stabilized by the rigid central peptide core residues Glu-3, Leu-4 & Ala-5 (grey circle 2). No crystal contacts were made by the C-terminal hairpin loop (grey circle 3). HLA-DR1 represented as cartoon (green & cyan; symmetry mates = grey) with contacting side chains represented as sticks. Peptide is represented as sticks colored by B-factor (blue to red; B-factor range = 21 to 130).

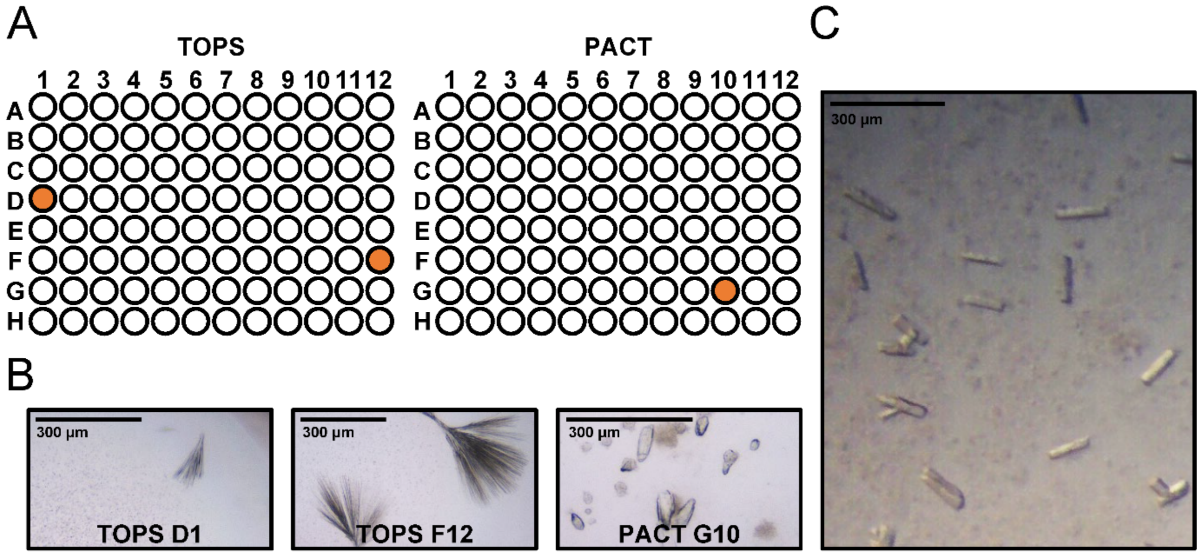

Figure S6. Optimized growth of HLA-DR1-5T4$_{111\text{-}130}$ protein crystals through microseeding. (*A*) Schematic overview of crystal condition well locations (TOPS D1, TOPS F12 & PACT G10) in which primary screens yielded non-diffractable crystalline material (orange) later used as microseeds for crystal optimization. (*B*) Stereomicroscope images highlighting non-diffractable crystal forms from primary crystal screening conditions as outlined in Fig. S5. *A*.(*C*) Stereomicroscope images of high-resolution diffracting 3-Dimensional HLA-DR1-5T4$_{111\text{-}130}$ crystals grown at 4 mg/mL via hanging drop in PACT G10 buffer condition (0.02 M Sodium/potassium phosphate, 0.1 M Bis-Tris propane pH 7.5, 20 % PEG 3350) supplemented with microseed stocks produced from combined crystals described previously. Obtained crystals were approximately 80 µm along the longest edge.

Video S1. MD trajectories illustrating peptide mobility. Backbone atoms of PFRs are shown as sticks and color-coded as indicated (inset). Core residues (showing all atoms) are blue sticks with atom coloring as previous. DRα (grey cartoon) and DR1β (purple cartoon) represented from the static crystallographic structure.
